# A Case Report on Abdominal Pain Treated With a New Technique of Ultrasound-Guided Transversus Abdominis Plane Hydrodissection Using a Low Concentration of Local Anesthetics

**DOI:** 10.7759/cureus.31966

**Published:** 2022-11-28

**Authors:** Mihiro Kaga

**Affiliations:** 1 Emergency and General Internal Medicine, Rakuwakai Marutamachi Hospital, Kyoto, JPN

**Keywords:** lateral cutaneous nerve entrapment syndrome, anterior cutaneous nerve entrapment syndrome, carnett's sign, transversus abdominis plane, ultrasound-guided, abdominal wall, spinal nerve, ventral ramus, hydrodissection

## Abstract

Hydrodissection, a technique for performing morphological dissection between the target tissues via injection, has attracted attention in recent years. However, high-quality evidence is available only for a few entrapment neuropathies, such as carpal tunnel syndrome, and further case studies are needed for other diseases. This case report presents the first case of hydrodissection of the ventral ramus of the spinal nerve, which innervates the abdominal wall, to improve abdominal pain. A 59-year-old Japanese man with a history of cerebral infarction and dyslipidemia presented to the emergency department with the chief complaint of left upper abdominal pain that began two days earlier. The pain radiated to the left side of the back and left axilla. The abdomen was flat and soft with no tenderness on examination, and the pinch test was negative. However, Carnett's sign was observed in the left upper abdomen, and the location of the left upper abdominal pain and that of the radiating pain were consistent with the ninth thoracic vertebra (Th9) dermatome; thus, the cause of the left upper abdominal pain was determined to be the ventral ramus of the spinal nerve of Th9. Two weeks of physical therapy and lifestyle guidance were ineffective; therefore, hydrodissection of the transversus abdominis plane (TAP) between the myofascia of the internal oblique muscle and that of the transversus abdominis at the Th9 level using a 23G 60 mm needle under ultrasound guidance was planned. The abdominal pain immediately improved after hydrodissection, and the patient was able to work without pain. Thus, ultrasound-guided TAP hydrodissection with a low-concentration local anesthetic is effective in the treatment of abdominal pain caused by the entrapment of the ventral ramus of the spinal nerve due to adhesions between the myofascia of the internal oblique muscle and that of the transversus abdominis. This condition should be termed ventral ramus of spinal nerve entrapment syndrome (VERNES), and this concept and TAP hydrodissection must be made known to the public.

## Introduction

Peripheral nerve blocks for paresthesia and analgesia are frequently used in operating rooms and pain clinics. Hydrodissection, an injection technique seemingly similar to peripheral nerve blocks, has been attracting attention in recent years [1]. The development of ultrasound equipment has enabled precise injection into the target area, and both techniques are used increasingly. Peripheral nerve block induces paresthesia by infiltrating a local anesthetic around the target nerve and requires a local anesthetic as the injection solution [2]. In contrast, hydrodissection aims for morphological dissection between the target tissues, and the injectable solution does not necessarily require a local anesthetic [1,3]. The two techniques serve different purposes. Clinical studies on the efficacy of hydrodissection have shown a high level of evidence for carpal tunnel syndrome [4]; however, only a few reports of improvement in pain with hydrodissection are available [5,6], and further case studies are needed to verify its efficacy. The anterior abdominal wall is innervated by the seventh thoracic vertebra to the first lumbar vertebra (Th7-L1) spinal nerves [7], with the ventral ramus of the spinal nerves running in the transversus abdominis plane (TAP) between the myofascia of the internal oblique muscle and that of the transversus abdominis [8]. The ventral ramus of the spinal nerve branches off the lateral cutaneous nerve at the level of the midaxillary line and the anterior cutaneous nerve at the outer border of the rectus abdominis muscle [9]. There have been no case reports on the treatment of abdominal pain by hydrodissection of the ventral ramus of the spinal nerve. This case report presents the first case of hydrodissection of the ventral ramus of the spinal nerve, which controls abdominal wall sensation, to improve abdominal pain.

## Case presentation

The patient was a 59-year-old Japanese man with a more than 10-year history of cerebral infarction and dyslipidemia. He had no physical disability from the stroke. He was receiving aspirin, pitavastatin calcium hydrate, irbesartan, and amlodipine besylate. He had no history of smoking but drank about 350 mL of canned beer once a week. The patient worked at a dyeing factory. His work was strenuous and required lifting and carrying an 18-L square can by hand and agitating liquids in a large bathtub while bending forward. He often experienced left upper abdominal pain for about 30 minutes at work, and the sensation was similar to a muscle being squeezed in the abdomen. The maximum pain rating was 8/10 on the numerical rating scale (NRS), and the patient was unable to move. The pain radiated to the left side of the back and left axilla. Occasionally, the pain was perceived even at rest. The pain was often relieved on nonworking days. Vomiting, nausea, cold sweats, or chest pain was not present.

**Figure 1:**
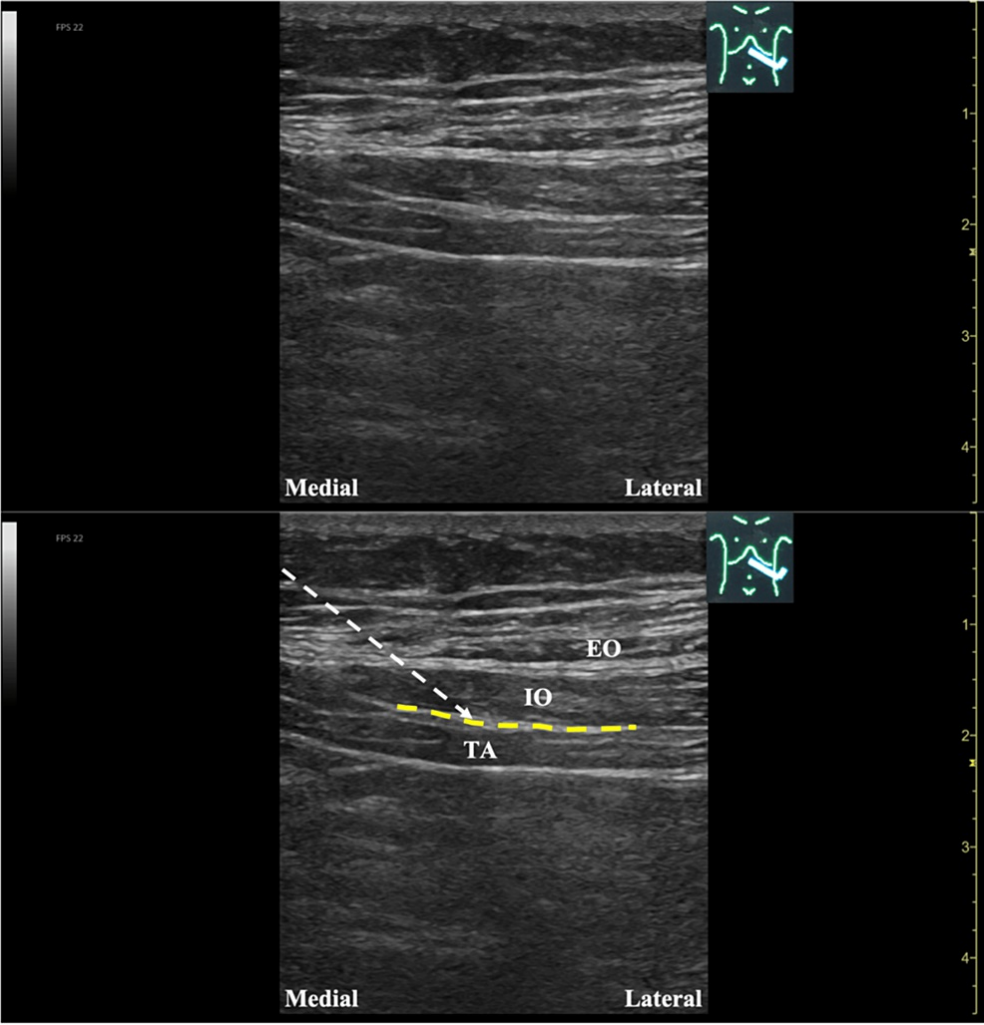
Ultrasound image at the time of puncture (the modality used was Venue GO R2 Final, GE Healthcare Japan Corporation, Tokyo, Japan). The white dashed line shows the needle trajectory. TA, transversus abdominis; IO, internal oblique muscle; EO, external oblique muscle; TAP, transversus abdominis plane (yellow dashed line)

The vital signs at presentation were as follows: body temperature 36.5 °C, blood pressure 115/68 mmHg, heart rate 72 beats/minute, and saturation of oxygen 99% (room air). Physical examination revealed no significant findings related to the head, neck, chest, or extremities. There was no weakness in the extremities. The abdomen was flat and soft with no tenderness, there were no tender points on the body surface, and the pinch test was negative. Guarding was not present. However, Carnett's sign was positive [10], and the location of the left upper abdominal pain and that of the radiating pain coincided with the Th9 dermatome. No rash is observed at the site of pain or elsewhere. The left upper abdominal pain was determined to have originated from the ventral ramus of the Th9 spinal nerve [7,8]. As the abdominal pain was induced during physical exertion using abdominal muscles, the ventral ramus of the spinal nerve running in the TAP between the internal oblique muscle and the transversus abdominis may have been entrapped by the adhesion between these myofascia. Physical therapy and lifestyle guidance, including working posture, were recommended, and 500 mg of acetaminophen orally up to four times daily, as needed, was prescribed for pain. The patient returned for a follow-up visit two weeks later. As the abdominal pain showed no improvement at the time of the return visit and acetaminophen had not been effective in managing abdominal pain, it was necessary to directly release the ventral ramus of the spinal nerve from where it was entrapped. An ultrasound-guided hydrodissection was planned for diagnostic and therapeutic purposes. Ultrasound-guided hydrodissection of the TAP between the myofascia of the internal oblique muscle and that of the transversus abdominis in the left upper abdomen at the Th9 level (TAP hydrodissection) was performed using a 23G 60 mm needle and low-concentration local anesthetic (1% lidocaine 1 mL + saline 9 mL). The needles were punctured using the parallel method. The ultrasonographic anatomy at the time of puncture is shown in Figure 1. Ultrasonography of both short-axis and long-axis images revealed no difference in the thickening of the myofascia between the internal oblique muscle and that of the transversus abdominis between the right and left sides. An ultrasonographic examination of the abdominal cavity and body surface failed to reveal any other cause of the left upper abdominal pain. Immediately after the injection, Carnett's sign and abdominal pain disappeared, but the abdominal pain (NRS 3/10) returned that evening. However, the abdominal pain receded by the next day, and the patient was able to perform his job without problems the day after the first injection and never experienced a recurrence of abdominal pain after the first injection. The patient was reviewed two weeks after the first injection, and no new symptoms appeared. Because of the patient's request, a second hydrodissection was performed on the same day, and the patient was followed up for 28 days after the first injection, with no recurrence of abdominal pain.

## Discussion

This case report is the first to adopt ultrasound-guided TAP hydrodissection using a low-concentration local anesthetic for the entrapment of the ventral ramus of the spinal nerve due to adhesion of TAP between the myofascia of the internal oblique muscle and that of the transversus abdominis.

The left upper abdominal pain was determined to have originated from the ventral ramus of the spinal nerve based on the physical examination, medical history, and ultrasound findings. Carnett’s sign, which was observed in this case, is a method for determining whether the pain originates from the abdominal wall or cavity [10]. The patient was placed in a supine position, with the head slightly elevated and the abdominal muscles tensed, and palpation was performed. If the pain persists or worsens, it may be originating from the abdominal wall (muscles, bones, or nerves); if the pain lessens, it may be due to intra-abdominal disease [11]. As the location of the abdominal pain and that of the radiating pain coincided with the Th9 dermatome, it was considered that the nerve causing the pain originated from the spinal nerve. Carnett's sign can be positive even in the absence of abdominal tenderness, as in this case, and the clinician should evaluate for Carnett's sign even in the absence of tenderness on palpation of the abdomen [12]. The pinch test checks for disproportionate pain by pinching the skin around the tender point. It is effective in diagnosing anterior cutaneous nerve entrapment syndrome (ACNES) with entrapment of the anterior cutaneous nerve and lateral cutaneous nerve entrapment syndrome (LACNES) with entrapment of the lateral cutaneous nerve [13,14]. The pinch test was negative in this case, and shallow palpation of the abdomen revealed no tenderness on the body surface, suggesting that the problem was at the level of the ventral ramus of the spinal nerve, not a superficial nerve problem at the level of the anterior cutaneous nerve or lateral cutaneous nerve of the spinal nerve. Ultrasound examination was also performed to rule out intra-abdominal lesions or other superficial body surface disease, and no abnormal findings were observed. Thickening of the myofascia can sometimes be observed on ultrasound if the nerve is entrapped by the thickening of the myofascia [15]; however, this was not the case in this patient. In addition, ultrasonographic evaluation of fascial gliding during the movement of the muscles may be useful in evaluating the pathophysiology of entrapment neuropathy [5]; however, this was not known in this case. Thus, diseases of muscular, bony, or nerve origin of the abdominal wall are often not accompanied by abnormalities on examination and cannot be diagnosed without a careful history and physical examination, as in this case. Injection therapy is an important diagnostic treatment for ANCES and LACNES [16,17]. Hydrodissection was used in this case, which was successful, suggesting that the ventral ramus of the spinal nerve was entrapped by TAP between the myofascia of the internal oblique muscle and that of the transversus abdominis.

TAP block is a technique similar to TAP hydrodissection and is used commonly in anesthesiology for peripheral nerve block of the ventral ramus of the spinal nerve running through TAP [1,3]. Peripheral nerve blocks aim to achieve paresthesia by infiltrating a local anesthetic around the target nerve, and the injection solution must be a local anesthetic, whereas hydrodissection aims for morphological detachment between the target tissues, and the injection solution does not necessarily require a local anesthetic [1]; thus, the two techniques serve different purposes. In the present case, pathophysiology was considered to be physical entrapment by the structures around the traveling nerve; therefore, TAP hydrodissection was performed instead of TAP block. A low-concentration local anesthetic (1% lidocaine 1 mL + saline 9 mL) was used for TAP hydrodissection in this case. Saline alone without local anesthetics would be effective. However, saline injections alone are not covered by insurance in Japan; therefore, a low-concentration local anesthetic was used. Unlike peripheral nerve blocks, hydrodissection does not require local anesthetics and can significantly reduce the risk of adverse events, such as continued numbness, pins and needles sensation, local anesthetic poisoning, and drug allergy [3]. In addition, certain patients may experience rebound pain after a peripheral nerve block, in which pain increases as the effect of the peripheral nerve block wears off, and pain control may be difficult [18]. However, this is not a concern with hydrodissection, as it does not require local anesthetics [3]. Common precautions for peripheral nerve block and hydrodissection include nerve damage and vascular damage [19].

An extension tube was attached between the 23G 60 mm needle and syringe as the injection tool in this case, and the person performing the injection held the needle and asked an assistant to hold the syringe and inject the solution. By using an assistant and attaching the extension tube, the distance from the injection site to the hand was shortened than that when one person was to hold the syringe with the needle and inject alone. We believe that doing so improves the manipulation of the needle.

Some patients with neuropathy due to entrapment of the ventral ramus of the spinal nerve, as in this case, remain undiagnosed. This condition should be termed ventral ramus of spinal nerve entrapment syndrome (VERNES), and this concept and TAP hydrodissection must be made known to the public. In recent years, cases of hydrodissection treatment for nerve entrapment have been reported in various diseases, such as piriformis syndrome [5], occipital neuralgia [6], and dorsal scapular nerve entrapment neuropathy [20], and hydrodissection may be successful in treating nerve entrapment between the myofascia that has not been reported elsewhere.

## Conclusions

The symptoms caused by entrapment of the ventral ramus of the spinal nerve due to adhesions between the myofascia of the internal oblique muscle and that of the transversus abdominis are known as VERNES. Ultrasound-guided TAP hydrodissection with a low concentration of a local anesthetic may be effective in the treatment of VERNES. Hydrodissection may also be successful in treating other types of unreported inter-myofascial nerve entrapment, and further case reports and larger scale studies are needed.
